# Description of vaccination coverage and hesitancy obtained by epidemiological survey of children born in 2017-2018, in Belo Horizonte and Sete Lagoas, Minas Gerais, Brazil

**DOI:** 10.1590/S2237-96222024v33e20231188.especial2.en

**Published:** 2024-08-23

**Authors:** Taynãna César Simões, Orozimbo Henriques Campos, Ana Paula França, José Cássio de Moraes, ICV 2020 Group, Adriana Ilha da Silva, Alberto Novaes Ramos, Ana Paula França, Andrea de Nazaré Marvão Oliveira, Antonio Fernando Boing, Carla Magda Allan Santos Domingues, Consuelo Silva de Oliveira, Ethel Leonor Noia Maciel, Ione Aquemi Guibu, Isabelle Ribeiro Barbosa Mirabal, Jaqueline Caracas Barbosa, Jaqueline Costa Lima, José Cássio de Moraes, Karin Regina Luhm, Karlla Antonieta Amorim Caetano, Luisa Helena de Oliveira Lima, Maria Bernadete de Cerqueira Antunes, Maria da Gloria Teixeira, Maria Denise de Castro Teixeira, Maria Fernanda de Sousa Oliveira Borges, Rejane Christine de Sousa Queiroz, Ricardo Queiroz Gurgel, Rita Barradas Barata, Roberta Nogueira Calandrini de Azevedo, Sandra Maria do Valle Leone de Oliveira, Sheila Araújo Teles, Silvana Granado Nogueira da Gama, Sotero Serrate Mengue, Taynãna César Simões, Valdir Nascimento, Wildo Navegantes de Araújo

**Affiliations:** 1 Instituto René Rachou, Núcleo de Estudos em Saúde Pública e Envelhecimento, Belo Horizonte, MG, Brasil; 2 Universidade Federal de Minas Gerais, Faculdade de Medicina, Belo Horizonte, MG, Brasil; 3 Faculdade de Ciências Médicas da Santa Casa de São Paulo, Departamento de Saúde Coletiva, São Paulo, SP, Brasil; 4 Universidade Federal do Espírito Santo, Vitória, ES, Brazil; 5 Universidade Federal do Ceará, Departamento de Saúde Comunitária, Fortaleza, CE, Brazil; 6 Faculdade Ciências Médicas Santa Casa de São Paulo, São Paulo, SP, Brazil; 7 Secretaria de Estado da Saúde do Amapá, Macapá, AP, Brazil; 8 Universidade Federal de Santa Catarina, SC, Brazil; 9 Organização Pan-Americana da Saúde, Brasília, DF, Brazil; 10 Instituto Evandro Chagas, Belém, PA, Brazil; 11 Faculdade de Ciências Médicas Santa Casa de São Paulo, Departamento de Saúde Coletiva, São Paulo, SP, Brazil; 12 Universidade Federal do Rio Grande do Norte, Natal, RN, Brazil; 13 Universidade Federal do Ceará, Programa de Pós-Graduação em Saúde Pública, Fortaleza, CE, Brazil; 14 Universidade Federal de Mato Grosso, Cuiabá, MT, Brazil; 15 Universidade Federal do Paraná, Curitiba, PR, Brazil; 16 Universidade Federal de Goiás, Goiânia, GO, Brazil; 17 Universidade Federal do Piauí, Teresina, PI, Brazil; 18 Universidade de Pernambuco, Faculdade de Ciências Médicas, Pernambuco, PE, Brazil; 19 Instituto de Saúde Coletiva, Universidade Federal da Bahia, Salvador, BA, Brazil; 20 Secretaria de Estado da Saúde de Alagoas, Maceió, AL, Brazil; 21 Universidade Federal do Acre, Rio Branco, AC, Brazil; 22 Universidade Federal do Maranhão, Departamento de Saúde Pública, São Luís, MA, Brazil; 23 Universidade Federal de Sergipe, Aracaju, SE, Brazil; 24 Secretaria Municipal de Saúde, Boa Vista, RR, Brazil; 25 Fundação Oswaldo Cruz, Mato Grosso do Sul, Campo Grande, MS, Brazil; 26 Fundação Oswaldo Cruz, Escola Nacional de Saúde Pública Sergio Arouca, Rio de Janeiro, RJ, Brazil; 27 Universidade Federal do Rio Grande do Sul, Porto Alegre, RS, Brazil; 28 Fundação Oswaldo Cruz, Instituto de Pesquisa René Rachou, Belo Horizonte, MG, Brazil; 29 Secretaria de Desenvolvimento Ambiental de Rondônia, Porto Velho, RO, Brazil; 30 Universidade de Brasília, Brasília, DF, Brazil

**Keywords:** Cobertura de Vacunación, Encuestas Epidemiológicas, Programas de Inmunización, Calendario Infantil Básico, Disparidades Socioeconómicas en Salud, Vacilación ante las Vacunas, Vaccination Coverage, Health Surveys, Immunization Programs, Basic Children’s Schedule, Socioeconomic Disparities in Health, Vaccination Hesitancy

## Abstract

**Objective:**

To describe vaccination coverage and hesitation for the basic children’s schedule in Belo Horizonte and Sete Lagoas, Minas Gerais state, Brazil.

**Methods:**

Population-based epidemiological surveys performed from 2020 to 2022, which estimated vaccine coverage by type of immunobiological product and full schedule (valid and ministered doses), according to socioeconomic strata; and reasons for vaccination hesitancy.

**Results:**

Overall coverage with valid doses and vaccination hesitancy for at least one vaccine were, respectively, 50.2% (95%CI 44.1;56.2) and 1.6% (95%CI 0.9;2.7), in Belo Horizonte (n = 1,866), and 64.9% (95%CI 56.9;72.1) and 1.0% (95%CI 0.3;2.8), in Sete Lagoas (n = 451), with differences between socioeconomic strata. Fear of severe reactions was the main reason for vaccination hesitancy.

**Conclusion:**

Coverage was identified as being below recommended levels for most vaccines. Disinformation should be combated in order to avoid vaccination hesitancy. There is a pressing need to recover coverages, considering public health service access and socioeconomic disparities.

## Study contributions

**Main results:**

Vaccination coverage of children up to 4 years old was 50.2% in Belo Horizonte, and 64.9% in Sete Lagoas. Fear of severe reactions and believing that vaccination against eradicated diseases is unnecessary were the main reasons for vaccination hesitancy.

**Implications for services:**

Recovery of high vaccination coverage among children, considering public health service access conditions and socioeconomic inequities. Acting on reasons for hesitancy that can assist in targeting actions.

**Perspectives:**

The multifactorial context of vaccination hesitancy demands the development of health education strategies to raise awareness about child immunization.

## RESUMEN

**Objetivo:**

Descrever as coberturas e vacilación de las vacunas del calendario básico infantil en Belo Horizonte y Sete Lagoas, Minas Gerais.

**Métodos:**

Inquéritos epidemiológicos de base poblacional realizados, de 2020 a 2022, para estimar coberturas vacunales por tipo de imunobiológico y esquema completo (dosis válidas y aplicadas) segundos estratos socioeconómicos, y los motivos de vacilación vacunal.

**Resultados:**

A cobertura vacunale global con dosis válidas e a vacilación vacunal de pelo menos uma vacuna foram, respectivamente, de 50,2% (IC_95_% 44,1;56,2) e 1,6% (IC_95_% 0,9; 2,7), en Belo Horizonte (n = 1.866), e de 64,9% (IC_95_% 56,9;72,1) y 1,0% (IC_95_% 0,3;2,8), en Sete Lagoas (n = 451), con diferencias entre los estratos. O medo de reações graves foi o principal motivo de vacilación.

**Conclusión:**

Identificou-se cobertura vacunale abaixo do preconizado para a mayoria das vacunas. La desinformación debe ser combatida, evitando la vacilación vacunal. Há necessidad de recuperar la cobertura vacunale, considerando el acesso y las disparidades socioeconómicas.

## INTRODUCTION

The impacts of vaccines on the quality of life and longevity of modern society, promoting general health well-being throughout the world, are undeniable.^1^ Although the action of the National Immunization Program (PNI) has been highly successful in preventing important infectious parasitic diseases over the years in Brazil, vaccination coverage has shown considerable drops since 2016.^2-4^ In particular, the BCG vaccine (bacillus Calmette-Guérin) showed a 10% drop in the period.^5^

This phenomenon is multifactorial and may be related to problems of underrecording doses administered, outdated population estimates, influence of fake news, anti-vaccine movements, lack of access to Brazilian National Health System (SUS) primary care services, shortage of immunobiological products, vulnerable socioeconomic conditions, vaccination hesitancy, among other factors.^2-4^

Routine vaccination undertaken by the PNI establishes a national schedule that should apply to each individual from birth, in order to guarantee specific prevention against certain vaccine-preventable diseases, aiming to induce mass or herd immunity, with the aim of interrupting transmission or maintaining levels that have low potential to generate epidemics of emerging and re-emerging diseases.^1,6-8^

With the aim of supporting control and prevention actions, epidemiological surveillance aims to obtain accurate and timely information about coverages. However, estimates, based on indicators the denominators of which may be overestimated or underestimated, may distort results and lead to coverages being considered adequate, when in fact it is insufficient to achieve collective protection and prevent circulation of etiological agents.^8-9^

Within this context, the National Vaccination Coverage Survey 2020 (INCV 2020) aimed to compute coverage in a more realistic way, and this study aimed to describe vaccination coverage and hesitancy for the basic childhood schedule based on the INCV surveys carried out in Belo Horizonte and Sete Lagoas.

## METHODS

### Study design

This is a population-based survey, carried out in the cities of Belo Horizonte and Sete Lagoas, between September 2020 and March 2022. The study is part of the INCV 2020 carried out in the Brazilian state capitals, Federal District and in 12 cities in the interior region of Brazil with more than 100,000 inhabitants.^3,10^

### Background

In 2020, Belo Horizonte had an estimated resident population of 2,521,564 inhabitants, 5.2% (130,707) of whom were children born into the 2017 and 2018 cohorts, and a birth rate of 10.42 live births per 1,000 inhabitants. According to the PNI Information System, in 2018 there were 191 vaccination rooms that were either public, private or both. In addition to the state capital Belo Horizonte, we chose the city of Sete Lagoas from among municipalities located outside the Belo Horizonte metropolitan region with more than 100,000 inhabitants because its coverage is one of the lowest. In 2020 the municipality of Sete Lagoas had an estimated resident population of 241,835 inhabitants, 5.9% (14,167) of whom children born into the 2017-2018 cohorts, and a birth rate of 10.70 live births per 1,000 inhabitants. Taking both the public and private health sectors, 28 establishments administered vaccines in 2021, according to data from the Sete Lagoas City Health Department.^10^

### Participants 

The target population was comprised of 59,957 live births in Belo Horizonte and 5,261 live births in Sete Lagoas from the 2017-2018 birth cohorts.

### Sampling

The sampling procedure was carried out in multiple stages. The stratified sample, according to socioeconomic strata, was formed by clusters with selection in two stages (random selection of census tracts and households). 

In each municipality, the socioeconomic strata were defined by ordering the census sectors according to the average income of those responsible for each household, the proportion of literate child guardians and income greater than or equal to 20 minimum wages. The census tracts were the primary units of analysis.

In Belo Horizonte, stratum A (high) was composed of 183 tracts (Sete Lagoas = 42), with average monthly income expressed in minimum wages (MW) of 18.21% (Sete Lagoas = 6.69%), 99.8% (Sete Lagoas = 98.3%) literate people, and 26.5% (Sete Lagoas = 4.4%) with income > 20 MW. Stratum B (medium) with 294 tracts (Sete Lagoas = 42), 10.50% MW (Sete Lagoas = 3.18%), 99.9% (Sete Lagoas = 97.3%) and 11.1% (Sete Lagoas = 0.9%), respectively. Stratum C (low) with 1,091 tracts (Sete Lagoas = 69), 5.19% MW (Sete Lagoas = 2.23%), 99.4% (Sete Lagoas = 95.7%) and 2.4% (Sete Lagoas = 0, 3%). Stratum D (very low) with 2,262 tracts (Sete Lagoas = 126), 1.91% MW (Sete Lagoas = 1.54%), 94.0% (Sete Lagoas = 92.4%) and 0.2% (Sete Lagoas = 0.1%).

Subsequently, clusters were defined with a minimum number of children to be selected randomly, in order to reach the desired sample size. The children were located based on the geographic coordinates of the addresses available on the Live Birth Information System and, when necessary, by active searching in the clusters. Details of sampling in each municipality have already been described in previous publications.^3,10^

### Variables of interest

The full basic schedule included the set of vaccines to be administered up to 24 months of life and which are included in the Ministry of Health’s basic childhood vaccination schedule: BCG, hepatitis B, 5-in1, inactivated poliovirus vaccine (IPV), 10-valent pneumococcal conjugate, human rotavirus, meningococcal C conjugate, yellow fever. The full schedule at 24 months includes, in addition to the basic schedule vaccines, MMR (measles, mumps and rubella), hepatitis A, chickenpox and attenuated oral poliovirus vaccine (OPV), DTP booster (diphtheria, pertussis and tetanus), meningococcal C and pneumococcal vaccine.^3^ Yellow fever vaccine was not included because its having been introduced or not into the basic schedule varied between states.

The exposure variables were sociodemographic, maternal (reproductive), household, family consumption, and child vaccination data, in addition to reasons for vaccination hesitancy, difficulties encountered, and guardians’ perceptions about vaccines.^3^ As different compositions of vaccines are used to protect against the same diseases and as their use is different in the public and private sectors, in these situations, the administration dates of these vaccines were standardized in a variable related to each vaccine on the PNI schedule, such as 5-in-1 vaccine = 5-in-1 + hexavalent + acellular. Details of the procedure for each vaccine are described in the national survey technical report.^10^

### Data source/measurement

Coverage, using the administrative method, which represents the proportion of the target population vaccinated, was obtained by dividing the number of administered doses of a vaccine by the target population, multiplied by 100.^11^ In the survey, coverage was calculated based on the administration dates of vaccines recorded on vaccination cards, with vaccination schedules being calculated for administered doses (number of recorded doses of each vaccine) and valid/timely doses (considering the time they were administered in relation to date of birth and intervals between doses).^3^ These data were obtained from photographs of the vaccination cards, which were interpreted and transcribed by professionals with vaccination room experience. Children without a vaccination card were considered unvaccinated after an unsuccessful search for their record on the PNI Information System. Further information was obtained via the questionnaire answered by the person responsible for each child.

Details of the field work, data collection and transcription, as well as problems noted and potential biases, have already been presented in previous publications.^3,10^

### Variable definition and categorization

Sociodemographic characteristics of the families:

- Family consumption level: defined according to cutoff points of the 2019 Brazilian Economic Classification Criteria: high (42 points and more), medium (27 to 41 points), low (16 to 26 points) and very low (< 16 points);^12^
- Household crowding (recorded on the household questionnaire): more than three dwellers sharing a room used as a bedroom;
- Proportion of families benefitted by the Bolsa Família cash transfer program;
- Monthly family income categorized into brackets: no income or income up to BRL 300; BRL 301 - BRL 1000; BRL 1001 - BRL 3000; BRL 3001 - BRL 5000; BRL 5001 - BRL 8000; more than BRL 8000.

Maternal characteristics:

- Schooling (incomplete elementary, complete elementary or incomplete high school, complete high school or incomplete higher education, complete higher education or above);
- Age group (< 20 years, 20 - 34 years, ≥ 35 years), race/skin color (White, mixed race, Black, Asian, Indigenous), paid work (yes/no), lives with a partner (yes/no), number of children alive.

Child’s characteristics:

- Sex (male/female);
- Birth order (first, second, third, fourth or more);
- Race/skin color (White, mixed race, Black, Asian, Indigenous);
- Attends daycare (yes, no).

Parental perception regarding statements about vaccines was assessed using a Likert scale, with answer scores ranging from 1 (totally disagree) to 5 (totally agree). The score obtained was later regrouped into the following categories: I totally or partially disagree, I am indifferent, I totally or partially agree.^10^ Regarding vaccination hesitancy of those responsible for the child, agreements or disagreements in relation to the following statements were considered: vaccines are not important; does not trust vaccines provided by the government; does not believe that vaccines are important for children’s health; Vaccinations are not important for neighborhood children; there is no need for vaccines for diseases that no longer exist; vaccines cause severe reactions.

### Statistical methods

The descriptive analysis of coverages was carried out separately for Belo Horizonte and Sete Lagoas, by calculating summary statistical measures (means and proportions) and building graphs of point and interval estimates of coverages prevalence and other characteristics of the study population, considering the complex sampling plans, measurement weights and subsequent calibration of population samples.^3,10^ The results were interpreted based on the coverage targets established by the Ministry of Health: 90%, for BCG and rotavirus; 95%, for hepatitis B, meningococcal C, 5-in-1, pneumococcal, poliomyelitis, hepatitis A, MMR; and 100% for DPT.

Estimates of the socioeconomic and demographic characteristics of the survey families, mothers and children were presented only for the highest and lowest income strata (A and D), due to similarities in coverage prevalence levels between the intermediate strata. The analyses were carried out using R statistical software version 4.3.2.^13^

### Ethical considerations

The study was approved by the Human Research Ethics Committee of the Public Health Institute of the Federal University of Bahia, under opinion number 3,366,818, on June 4, 2019, with Certificate of Presentation of Ethical Appreciation (CAAE) 4306919.5. 0000.5030; and the Irmandade da Santa Casa de São Paulo, under opinion number 4.380.019, on November 4, 2020, with CAAE 39412020.0.0000.5479. Interviewees signed a consent form for the interview and an authorization form for vaccine cards that were photographed.^3^

## RESULTS

We studied data on 1,866 children from Belo Horizonte (no losses), 470 from socioeconomic stratum A, 458 from stratum B, 469 from stratum C and 469 from stratum D. Of the total sample, 99.2% had a vaccination card. By strata, 99.9%, 98.5%, 99.0% and 99.4% of children, respectively, from strata A, B, C and D, had a vaccination card. 63.5% of children in stratum A and 9.3% in stratum D were vaccinated in private vaccination services. 

We studied data on 451 children from Sete Lagoas (no losses), 99.9% of whom had a vaccination card, with little variation between strata. Percentage of use of private vaccination services was 53.7% for stratum A, and 19.0% for stratum D. 

Table 1 shows the socioeconomic and demographic characteristics of the survey families, mothers and children, by highest and lowest extreme income strata (A and D) in Belo Horizonte and Sete Lagoas. In Belo Horizonte, the percentage of children from families with high and medium consumption was 70.1% in stratum A, while those with low and very low consumption accounted for 87.8% in stratum D. In Sete Lagoas, the proportion of families with high and medium consumption in stratum A (53.1%) was lower than that found for Belo Horizonte.

**Table 1:** Sociodemographic characteristics of the families, mothers and children included in the survey, by highest and lowest income strata, in Belo Horizonte and Sete Lagoas, National Vaccine Coverage Survey, Brazil, 2020

**Characteristics**	**Belo Horizonte (%) (n = 1,866)**			**Sete Lagoas (%) (n = 451)**		
	**Stratum A**	**Stratum D**	**Total**	**Stratum A**	**Stratum D**	**Total**
**Family characteristics**						
**Level of family consumption**						
High	15.3	0.3	3.0	5.2	0.0	1.1
Medium	54.8	12.0	22.1	47.9	9.5	16.5
Low	26.0	50.3	45.6	37.3	44.1	44.1
Very low	3.9	37.5	29.3	9.7	46.4	41.4
**Household crowding**	0.2	4.6	4.1	1.1	6.8	4.8
**Bolsa família**	6.8	18.2	15.1	7.3	24.9	23.8
**Monthly family income**						
Up to BRL 1,000	4.4	30.0	21.7	5.2	25.6	24.9
BRL 1,001-3,000	11.0	43.0	37.8	34.1	56.3	46.9
BRL 3,001-8,000	21.0	13.5	20.4	22.7	7.7	11.5
Over BRL 8,000	32.4	1.6	6.1	26.8	0.0	4.4
**Grandmother living together**	14.9	32.2	28.1	29.8	23.9	25.4
**Maternal characteristics**						
**Schooling**						
Fundamental incomplete	0.7	7.1	6.8	3.9	4.7	4.1
Complete elementary/Incomplete high school	2.9	14.9	13.0	12.4	12.9	13.2
Complete high school/Incomplete higher educ.	19.2	61.4	48.7	21.3	74.1	62.2
Complete higher educ. and above	76.5	15.0	30.2	62.4	6.1	19.1
**Age group (years)**						
< 20	0.0	2.2	1.8	0.0	1.1	0.6
20 - 34	28.0	64.7	54.9	34.9	50.2	51.6
≥ 35	72.0	33.1	43.2	65.1	48.6	47.9
**Race/skin color**						
White	71.1	52.5	38.8	42.6	9.8	15.6
Mixed race	20.3	32.4	40.2	5.7	20.1	16.1
Black	4.8	11.8	14.8	50.5	59.5	60.9
Asian	2.7	2.0	3.8	1.3	6.3	5.0
Indigenous	0.0	0.00	1.2	0.0	4.3	2.4
**Paid work**	81.9	44.4	53.5	75.4	37.0	46.9
**Has a partner**	86.7	68.0	70.3	77.1	73.7	68.9
**Average number of children alive**	1.6	2.0	1.9	1.7	1.9	1.8
**Child’s characteristics**						
**Sex**						
Male	51.1	59.1	56.4	62.7	47.5	53.6
Female	48.9	40.9	43.6	37.3	52.5	46.4
**Birth order**						
First	59.6	47.2	51.6	60.3	47.7	50.0
Second	33.0	33.0	32.8	25.3	39.5	34.3
Third	6.2	14.9	11.2	13.6	10.7	12.7
Fourth or more	1.3	4.9	4.2	0.9	2.2	3.1
**Race/skin color**						
White	71.7	40.9	48.9	57.2	33.6	36.5
Mixed race	22.3	45.4	39.9	1.5	8.6	6.5
Black	4.4	11.8	9.7	40.9	44.9	50.0
Asian	1.5	1.0	0.9	0.4	11.1	6.1
Indigenous	0.0	0.6	0.3	0.0	1.8	0.9
**Attends daycare**	62.1	61.6	61.9	42.4	22.7	27.2

In Belo Horizonte, 53.4% ​​of families in stratum A had monthly income of more than BRL 3000, while in Sete Lagoas, income was below this amount for 81.9% of stratum D families. In Belo Horizonte and Sete Lagoas, respectively, 15.0% and 6.1% of mothers in socioeconomic stratum D had higher education or above, while 76.5% and 62.4% of mothers in stratum A had higher education or above. 15.1% of families in Belo Horizonte, and 23.8% in Sete Lagoas, were Bolsa Família program beneficiaries. 

More than 50% of mothers were between 20 and 34 years old, lived with their partner (Belo Horizonte: 70.3%; Sete Lagoas: 68.9%) and had an average of 1.8 to 1.9 children, with a high variation in the frequency of these characteristics between strata. In Belo Horizonte, the majority of mothers reported being of White race/skin color (48.9%), while half of those interviewed in Sete Lagoas (50.0%) reported being Black in both strata. The majority of women with paid work belonged to stratum A (Belo Horizonte: 81.9%; Sete Lagoas: 75.4%), with lower social vulnerability. Regarding birth order, around half of the children were firstborn (Belo Horizonte: 51.6%; Sete Lagoas: 50.0%). 61.9% and 27.2% of children attended daycare, in Belo Horizonte and Sete Lagoas, respectively

**Figure 1:**
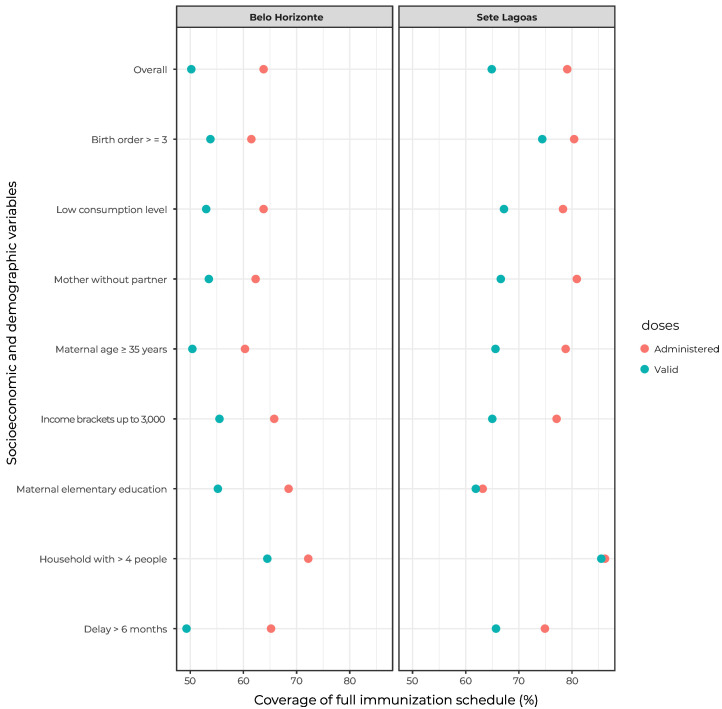
Coverage (%) of the full immunization schedule at 24 months, according to socioeconomic and demographic characteristics reported by the children’s guardians, in Belo Horizonte (n = 1,866) and Sete Lagoas (n = 451), National Vaccination Coverage Survey, Brazil, 2020

Figure 1 shows coverages point estimates (%) of the full schedule at 24 months of age, doses administered and valid doses, both total and according to population characteristics, in both municipalities. 

The municipality of Sete Lagoas had greater full schedule coverage (doses administered: 79.1 (95%CI 75.0;82.7); valid doses: 64.9 (95%CI 56.9;72.1) than Belo Horizonte (doses administered: 63.8 (95%CI 59.5;67.9); valid doses: 50.2 (95%CI 44.1;56.2)). In Sete Lagoas, coverage was higher among children living in households with four or more dwellers, with no difference between valid and administered doses. coverage of children whose mothers had completed elementary school was around 60% in Sete Lagoas.

Table 2 shows coverage of doses administered and valid doses for the immunobiological products assessed. In Belo Horizonte, coverage varied from 75.7% for the pneumococcal vaccine booster, to 90.3% for the first dose (D1) of 5-in-1 vaccine, with regard to valid doses.

**Table 2:** Average estimated vaccination coverage and 95% confidence interval (doses administered and valid doses) for each vaccine on the schedule for the first 24 months of life* in Belo Horizonte (n = 1,866) and Sete Lagoas (n = 451), National Vaccination Coverage Survey, Brazil, 2020

Vaccine	Coverage (doses administered) % (95%CI)		Coverage (valid doses) % (95%CI)	
	**Belo Horizonte**	**Sete Lagoas**	**Belo Horizonte**	**Sete Lagoas**
BCG	**↓**89.0 (83.3;92.9)	96.3 (88.0;98.9)	**↓**89.0 (83.3;92.9)	96.3 (88.0;98.9)
Hepatitis B	**↓**87.9 (82.4;91.9)	96.2 (88.1;98.8)	**↓**87.9 (82.4;91.9)	96.2 (88.1;98.8)
5-in-1 1^st^ dose	**↓**90.4 (84.6;94.2)	99.2 (98.1;99.7)	**↓**90.3 (84.5;94.1)	99.0 (97.7;99.5)
5-in-1 2^nd^ dose	**↓**89.8 (84.1;93.6)	96.3 (88.1;98.9)	**↓**89.7 (84.1;93.5)	96.3 (88.0;98.9)
5-in-1 3^rd^ dose	**↓**88.6 (83.2;92.4)	**↓**94.5 (87.9;97.6)	**↓**88.1 (82.8;92.0)	**↓**94.2 (87.7;97.4)
Polio 1^st^ dose	**↓**90.3 (84.6;94.1)	99.2 (98.1;99.7)	**↓**90.2 (84.5;94.0)	98.9 (97.6;99.5)
Polio 2^nd^ dose	**↓**89.7 (84.0;93.5)	96.3 (88.0;98.9)	**↓**89.6 (84.0;93.4)	95.9 (88.2;98.6)
Polio 3^rd^ dose	**↓**88.6 (83.2;92.4)	95.6 (88.3;98.4)	**↓**88.3 (82.9;92.1)	95.3 (88.2;98.2)
Pneumo 1^st^ dose	**↓**90.3 (84.5;94.0)	99.1 (97.9;99.6)	**↓**88.3 (82.4;92.5)	98.1 (96.3;99.0)
Pneumo 2^nd^ dose	**↓**89.8 (84.1;93.6)	96.3 (88.1;98.9)	**↓**89.7 (84.1;93.4)	95.9 (88.3;98.6)
Rotavirus 1^st^ dose	**↓**88.8 (83.3;92.7)	94.5 (87.5;97.7)	**↓**88.0 (82.6;91.9)	93.6 (86.9;97.0)
Rotavirus 2^nd^ dose	**↓**83.6 (78.4;87.7)	92.3 (86.2;95.8)	**↓**83.2 (78.1;87.3)	92.2 (86.1;95.7)
Meningitis C 1^st^ dose	**↓**89.9 (84.3;93.7)	99.3 (98.3;99.7)	**↓**89.2 (83.3;93.2)	99.3 (98.3;99.7)
Meningitis C 2^nd^ dose	**↓**89.4 (83.8;93.2)	96.2 (88.2;98.8)	**↓**88.8 (83.2;92.6)	**↓**89.4 (84.3;92.9)
Yellow fever	**↓**90.0 (84.3;93.8)	**↓**96.5 (87.9;99.1)	**↓**88.2 (82.5;92.2)	**↓**94.8 (88.0;97.8)
MMR 1^st^ dose	**↓**89.7(84.0;93.5)	96.7 (87.7;99.2)	**↓**88.9 (83.4;92.7)	96.4 (88.0;99.0)
MMR 2^nd^ dose	**↓**84.9 (79.7;88.9)	**↓**89.4 (83.2;93.5)	**↓**83.7 (78.7;87.7)	**↓**89.1 (83.0;93.2)
Hepatitis A	**↓**88.4 (83.0;92.3)	96.1 (88.2;98.8)	**↓**87.7 (82.4;91.6)	**↓**94.5 (88.1;97.5)
Chickenpox	**↓**88.0 (82.5;91.9)	**↓**92.1 (85.0;95.9)	**↓**84.8 (74.0;91.6)	**↓**91.1 (84.5;95.1)
Pneumo (boost.)	**↓**86.4 (81.3;90.3)	95.0 (88.1;98.0)	**↓**75.7 (71.1;79.8)	**↓**88.2 (82.7;92.1)
Meningitis C (boost.)	**↓**83.2 (78.2;87.3)	**↓**93.7 (87.6;96.9)	**↓**78.1 (73.0;82.4)	**↓**87.9 (82.6;91.8)
Polio (boost.)	**↓**84.2 (78.7;88.5)	95.7 (88.4;98.5)	**↓**81.3 (76.1;85.6)	**↓**91.3 (84.7;95.2)
DPT (boost.)	**↓**82.7 (77.8;86.8)	**↓**93.8 (87.9;96.2)	**↓**82.3 (77.4;86.3)	**↓**83.2 (78.7;87.0)
Full schedule*	63.8 (59.5;67.9)	79.1 (75.0;82.7)	50.2 (44.1;56.2)	64.9 (56.9;72.1)

BCG: bacillus Calmette-Guérin; DPT: diphtheria, pertussis and tetanus; meningitis C: meningococcal C; 5-in-1: diphtheria, tetanus, pertussis, hepatitis B and haemophilus influenzae type B; polio: poliomyelitis; pneumo: pneumococcal; MMR: measles, mumps and rubella; boost.: booster dose; 95%CI: 95% confidence interval; **↓** vaccination coverage below Ministry of Health recommendations: above 90% (BCG, rotavirus), above 95% (hepatitis B, polio, 5-in-1, meningitis C, hepatitis A, measles, mumps and rubella, chickenpox, polio booster), above 100% (yellow fever, DPT booster).

**Figure 2:**
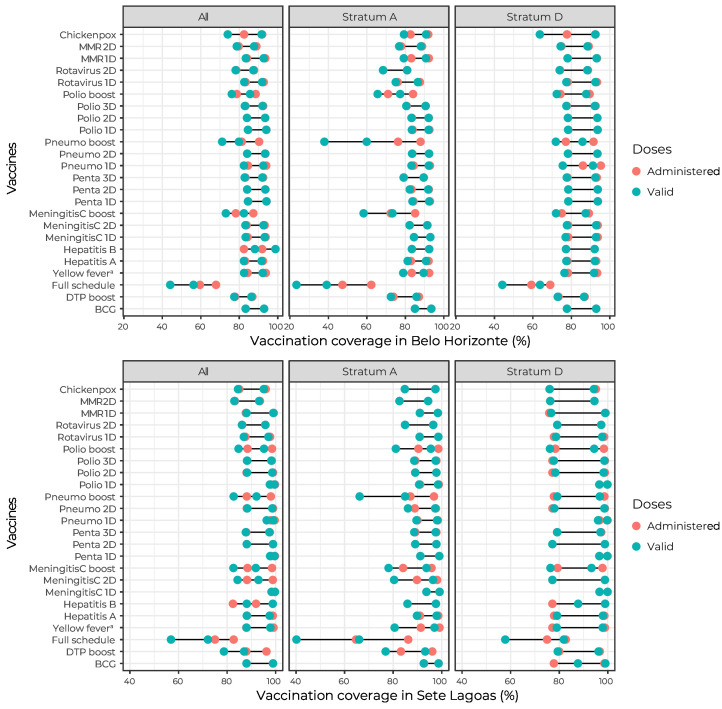
Vaccination coverage (doses administered and valid doses) on vaccines on the schedule of children up to 24 months old, for the total sample and by socioeconomic strata A and D, in Belo Horizonte (n = 1,866) and Sete Lagoas (n = 451), National Vaccination Coverage Survey, Brazil, 2020

Figure 2 shows coverage of the vaccines (administered and valid doses) on the vaccination schedule recommended by the Ministry of Health for children aged up to 24 months, in Belo Horizonte and Sete Lagoas. Coverage was lower in stratum A, although coverages variability was greater in stratum D in both municipalities. In Belo Horizonte, valid doses coverage was lower than that of doses administered for the pneumococcal vaccine booster, being higher in stratum A. In Sete Lagoas, there was a greater difference between doses administered and valid doses for the DPT booster in the general population, and for the pneumococcal booster in stratum A. 

Regarding vaccination hesitancy, among unvaccinated children, even in the absence of contraindications, families who decided not to vaccinate their children with one or more vaccines accounted for 1.6% (95%CI 0.9;2.7), in Belo Horizonte, and 1.0% (95%CI 0.3;2.8) in Sete Lagoas. The highest percentages of hesitancy, namely 1.6% (95%CI 0.7;3.6) in Belo Horizonte, and 1.2% (95%CI 0.2;6.1) in Sete Lagoas, occurred in stratum D. Difficulty accessing health centers was reported by 7.0% (95%CI 4.6;10.5) in Belo Horizonte, and by 6.1% (95%CI 3.8;9.5) in Sete Lagoas. Percentage vaccination hesitancy in strata A and D, respectively, was 3.5% (95%CI 1.1;11.0) and 8.7% (95%CI 5.1;14.5) in Belo Horizonte, and 5.1% (95%CI 2.2;11.0) and 2.6% (95%CI 0.9;7.2) in Sete Lagoas. 

Among the reasons for vaccination hesitancy, not vaccinating children, even when taking them for vaccination at a health center, was reported by 18.2% (95%CI 14.3;22.8) of guardians in Belo Horizonte. When analyzed by strata, hesitancy for this reason was 12.8% (95%CI 6.3;24.2) in stratum A and 18.9% (95%CI 13.3;26.2) in stratum D. In Sete Lagoas, this reason for hesitancy was reported by 33.1% (95%CI 28.2;38.3) of the children’s guardians, 34.5% (95%CI 22.6;48.7) from stratum A and 30.2% (95%CI 25.1;35.8) from stratum D. 

**Figure 3:**
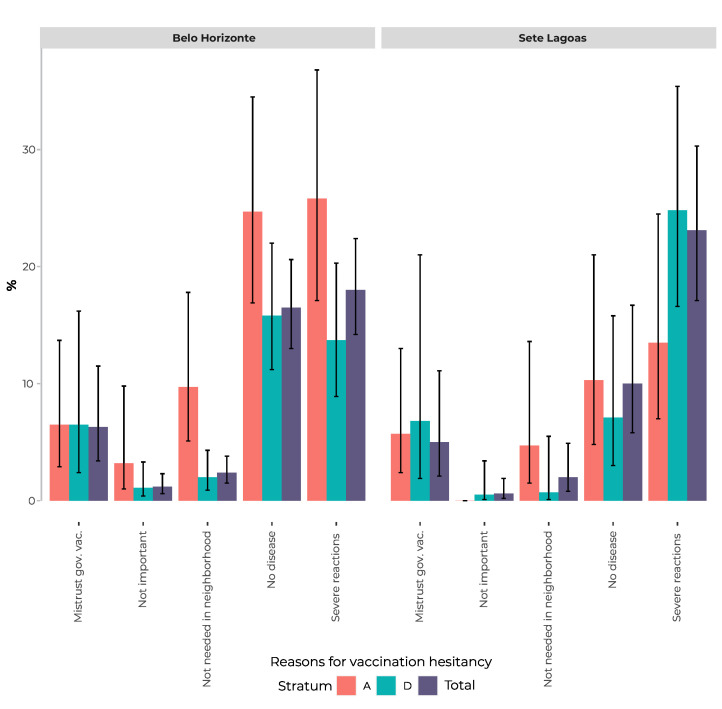
Reasons for vaccination hesitancy reported by the guardians of children up to 24 months old in Belo Horizonte (n = 1,866) and Sete Lagoas (n = 451), both total and by socioeconomic strata A and D, National Vaccination Coverage Survey, Brazil, 2020

Figure 3 shows the frequency of the main reasons for vaccination hesitancy, with emphasis on non-vaccination due to fear of severe reactions, which was reported by 18.0% (95%CI 14.2;22.4) of those responsible for child vaccination in Belo Horizonte, and by 23.1% (95%CI 17.1;30.3), in Sete Lagoas. Vaccination hesitancy due to the idea that it is unnecessary to vaccinate against diseases that no longer exist was reported by 16.5% (95%CI 13.0;20.6) of guardians interviewed in Belo Horizonte, and by 10.0% (95 %CI 5.8;16.7) in Sete Lagoas. The frequency distribution of reasons for hesitancy varied between economic strata, with the reasons for hesitancy mentioned above being most frequently reported by the children’s guardians in stratum A in Belo Horizonte, while in Sete Lagoas the most frequently reported reason for hesitancy in stratum D was fear of adverse reactions.

## DISCUSSION

In general, coverage was below recommended levels, with significant differences between the socioeconomic strata of the municipalities. In Belo Horizonte, all vaccines were below target, whereby rotavirus second dose and DPT and meningococcal C boosters had the lowest coverage in terms of doses administered, while rotavirus second dose and poliomyelitis and DPT boosters having the lowest coverage with valid doses. In Sete Lagoas, the MMR second dose and pneumococcal, meningococcal C and DPT boosters had the lowest coverage. 5-in-1 third dose, yellow fever, MMR second dose, chickenpox and meningococcal C and DPT boosters were below target. 

A considerable percentage of children’s guardians reported difficulties in accessing vaccination at the right time at their health centers. Other reasons for vaccination hesitancy cited were non-vaccination for fear of adverse events or the belief that it was unnecessary to vaccinate their children against diseases that no longer exist.

The results suggest good integration between immunization health services in relation to vaccination control for children, represented by the low proportion of children without vaccination cards. This may be related to strengthening linkage and trust in SUS health services, as well as social programs, such as the Bolsa Família program, requiring children to be vaccinated, a fact that generates greater awareness of the importance of vaccination in low-income communities and, consequently, higher coverages.^14,15^

Socioeconomic and demographic inequalities in the target population of municipalities can have an impact on children’s health, thus affecting coverages. Children in stratum D are more likely to belong to families with low income and low maternal education, as well as a greater number of children, making access to health services difficult and compromising fulfilment of the vaccination schedule. The lowest coverage occurred in the least vulnerable stratum (stratum A), despite there having been changes in social programs in the period, and the socioeconomic strata having been defined based on the 2010 Census. Furthermore, studies have shown that awareness of the importance of vaccination is higher in low income communities.^14,15^ Therefore, greater attention should be paid to stratum A, although also guaranteeing equitable access to vaccines in other strata, in order to protect the health and well-being of children.^16^

Coverage was higher in households with four or more dwellers, which may be explained by greater linkage with primary health services or coverage by the Family Health Strategy. In Sete Lagoas, coverage was lower among children whose mothers only had elementary education, possibly due to less access to information and education about the importance of immunization. 

Access can be enhanced through primary health care and the proximity of families to health services, as demonstrated by a systematic review in European countries and Australia. That 2019 study shows that structural and organizational aspects of health care systems for young children are important for equity in vaccine acceptance.^17^

There was greater vaccination hesitancy in stratum A in Belo Horizonte, possibly due to greater access to information and misinformation, such as fake news and rumors on digital media. Difficulty in accessing a primary health care center was the reason reported by families who were most vulnerable. Fear of severe reactions demands that information be disseminated about the real risks of vaccines, reducing misconceptions and promoting greater adherence.^18^ In this context, digital media amplify anti-vaccine discourse, with objections related to adverse events and minimization of disease severity.^19^

A global overview of systematic reviews on barriers to childhood vaccination identified 573 descriptions, categorized into six broad categories: (1) access, (2) clinical or health system barriers, (3) concerns and beliefs, (4) perceptions and experiences of health, (5) knowledge and information and (6) social or family influence. These reasons appeared in the INCV 2020, requiring reflection on strategies to change this scenario, such as awareness campaigns about the importance of vaccination, focused on strata with lower coverages, training of health professionals, improving access, with special attention to socially vulnerable families.^20^

Another systematic review assessed parents’ perceptions, showing that they considered mandatory immunization schedules to be a violation of their rights, and did not like schedules that offered financial incentives for vaccination. On the other hand, some parents felt that schedules limiting school access for unvaccinated children gave them peace of mind.^21^

Our study described information on vaccination coverage and hesitancy, in addition to official records, using information from public and private services, including information on unvaccinated children. Although it may have had an impact on the child immunization process, we did not assess the effects of the COVID-19 pandemic.^3,10^ A limitation of this study is that it was not possible to assess significant differences between the two municipalities studied, since the sample was not designed for this purpose. Both municipalities showed low coverages and inequalities between socioeconomic strata, pointing to the need to recover high coverages levels, prioritizing vaccines with coverage below recommended levels in all socioeconomic strata, considering greater access and health education.
