# Supplementary material for: Description of vaccination coverage and hesitancy obtained by epidemiological survey of children born in 2017-2018, in Belo Horizonte and Sete Lagoas, Minas Gerais, Brazil
Source: Epidemiol Serv Saude. 2024 Aug 23;33(spe2):e20231188. doi: 10.1590/S2237-96222024v33e20231188.especial2.en (PMC11351486; doi:10.1590/S2237-96222024v33e20231188.especial2.en)
Supplement: Translation

# Translation: Descrição da cobertura e da hesitação vacinal obtida por inquérito epidemiológico de crianças nascidas em 2017-2018, em Belo Horizonte e Sete Lagoas, Minas Gerais

**DOI:** 10.1590/S2237-96222024v33e20231188.especial2.pt

**Authors:** Taynãna César Simões, Orozimbo Henriques Campos, Ana Paula França, José Cássio de Moraes, Grupo ICV 2020

**Affiliations:** 1 Instituto René Rachou, Núcleo de Estudos em Saúde Pública e Envelhecimento, Belo Horizonte, MG, Brasil; 2 Universidade Federal de Minas Gerais, Faculdade de Medicina, Belo Horizonte, MG, Brasil; 3 Faculdade de Ciências Médicas da Santa Casa de São Paulo, Departamento de Saúde Coletiva, São Paulo, SP, Brasil; 4 Universidade Federal do Espírito Santo, Vitória, ES, Brasil; 5 Universidade Federal do Ceará, Departamento de Saúde Comunitária, Fortaleza, CE, Brasil; 6 Faculdade Ciências Médicas Santa Casa de São Paulo, São Paulo, SP, Brasil; 7 Secretaria de Estado da Saúde do Amapá, Macapá, AP, Brasil; 8 Universidade Federal de Santa Catarina, SC, Brasil; 9 Organização Pan-Americana da Saúde, Brasília, DF, Brasil; 10 Instituto Evandro Chagas, Belém, PA, Brasil; 11 Faculdade de Ciências Médicas Santa Casa de São Paulo, Departamento de Saúde Coletiva, São Paulo, SP, Brasil; 12 Universidade Federal do Rio Grande do Norte, Natal, RN, Brasil; 13 Universidade Federal do Ceará, Programa de Pós-Graduação em Saúde Pública, Fortaleza, CE, Brasil; 14 Universidade Federal de Mato Grosso, Cuiabá, MT, Brasil; 15 Universidade Federal do Paraná, Curitiba, PR, Brasil; 16 Universidade Federal de Goiás, Goiânia, GO, Brasil; 17 Universidade Federal do Piauí, Teresina, PI, Brasil; 18 Universidade de Pernambuco, Faculdade de Ciências Médicas, Pernambuco, PE, Brasil; 19 Instituto de Saúde Coletiva, Universidade Federal da Bahia, Salvador, BA, Brasil; 20 Secretaria de Estado da Saúde de Alagoas, Maceió, AL, Brasil; 21 Universidade Federal do Acre, Rio Branco, AC, Brasil; 22 Universidade Federal do Maranhão, Departamento de Saúde Pública, São Luís, MA, Brasil; 23 Universidade Federal de Sergipe, Aracaju, SE, Brasil; 24 Secretaria Municipal de Saúde, Boa Vista, RR, Brasil; 25 Fundação Oswaldo Cruz, Mato Grosso do Sul, Campo Grande, MS, Brasil; 26 Fundação Oswaldo Cruz, Escola Nacional de Saúde Pública Sergio Arouca, Rio de Janeiro, RJ, Brasil; 27 Universidade Federal do Rio Grande do Sul, Porto Alegre, RS, Brasil; 28 Fundação Oswaldo Cruz, Instituto de Pesquisa René Rachou, Belo Horizonte, MG, Brasil; 29 Secretaria de Desenvolvimento Ambiental de Rondônia, Porto Velho, RO, Brasil; 30 Universidade de Brasília, Brasília, DF, Brasil

**Keywords:** Cobertura Vacinal, Inquéritos Epidemiológicos, Programas de Imunização, Calendário Básico Infantil, Disparidades Socioeconômicas em Saúde, Hesitação Vacinal

## Abstract

**Objetivo:**

Descrever as coberturas e hesitação das vacinas do calendário básico infantil em Belo Horizonte e Sete Lagoas, Minas Gerais.

**Métodos:**

Inquéritos epidemiológicos de base populacional realizados de 2020 a 2022, para estimar coberturas vacinais por tipo de imunobiológico e esquema completo (doses válidas e aplicadas) segundo estratos socioeconômicos, e os motivos de hesitação vacinal.

**Resultados:**

A cobertura global com doses válidas e a hesitação vacinal de pelo menos uma vacina foram, respectivamente, de 50,2% (IC_95_% 44,1;56,2) e 1,6% (IC_95_% 0,9;2,7), em Belo Horizonte (n = 1.866), e de 64,9% (IC_95_% 56,9;72,1) e 1,0% (IC_95_% 0,3;2,8), em Sete Lagoas (n = 451), com diferenças entre os estratos. O receio de reações graves foi o principal motivo de hesitação vacinal.

**Conclusão:**

Identificou-se coberturas abaixo do preconizado para a maioria das vacinas. A desinformação deve ser combatida, evitando-se a hesitação vacinal. Há necessidade premente de recuperar as coberturas, considerando acesso ao SUS e disparidades socioeconômicas.

## Contribuições do estudo

**Principais resultados:**

A cobertura vacinal de crianças com até 24 meses de vida foi de 50,2% em Belo Horizonte, e 64,9% em Sete Lagoas. O receio de reações graves e a crença de que a vacinação contra doenças erradicadas é desnecessária foram os principais motivos de hesitação vacinal.

**Implicações para os serviços:**

Recuperar altas coberturas vacinais em crianças, considerando-se condições de acesso e iniquidades socioeconômicas. Atuar sobre os motivos de hesitação que podem auxiliar no direcionamento das ações.

**Perspectivas:**

O contexto multifatorial da hesitação vacinal demanda o desenvolvimento de estratégias de educação em saúde para promover a consciência para a imunização das crianças.

## MÉTODOS

### Desenho do estudo

Trata-se de um inquérito de base populacional, realizado nas cidades de Belo Horizonte e Sete Lagoas, entre setembro de 2020 e março de 2022. O estudo integra o INCV 2020, realizado nas capitais brasileiras, Distrito Federal, e em 12 cidades do interior do país com mais de 100 mil habitantes.^3,10^

### Contexto

Em 2020, Belo Horizonte tinha uma população residente estimada de 2.521.564 habitantes, sendo 5,2% (130.707) composta por crianças nascidas nas coortes de 2017 e 2018, e taxa de natalidade de 10,42 nascidos vivos por mil habitantes. Segundo o Sistema de Informações do PNI (SI-PNI), em 2018 existiam 191 salas de vacinas públicas, privadas, ou com ambas as estratégias. Além da capital, dos municípios fora da região metropolitana com mais de 100 mil habitantes, a cidade de Sete Lagoas foi escolhida por ter uma das menores coberturas. O município de Sete Lagoas tinha uma população residente estimada em 2020 de 241.835 habitantes, sendo 5,9% (14.167) de crianças nascidas nas coortes de 2017-2018, e taxa de natalidade de 10,70 nascidos vivos por mil habitantes. Somando-se os setores público e privado, 28 estabelecimentos aplicavam vacinas em 2021, segundo dados da Secretaria Municipal de Saúde do município.^10^

### Participantes 

A população-alvo foi de 59.957 nascidos vivos em Belo Horizonte e de 5.261 nascidos vivos em Sete Lagoas, das coortes de nascimento de 2017-2018.

### Amostragem

O procedimento de amostragem do inquérito nacional foi realizado em múltiplas etapas. A amostra estratificada, segundo estratos socioeconômicos, foi agrupada por conglomerados com seleção em dois estágios (seleção aleatória dos setores censitários e domicílios). 

Em cada município, os estratos socioeconômicos foram definidos a partir da ordenação dos setores censitários segundo a renda média dos responsáveis pelo domicílio, a proporção de responsáveis alfabetizados e renda maior ou igual a 20 salários mínimos. Os setores censitários foram as unidades primárias de análise.

Em Belo Horizonte, o estrato A (alto) foi composto por 183 setores (Sete Lagoas = 42), com renda média mensal em salários mínimos (SMs) de 18,21%, (Sete Lagoas = 6,69%), 99,8% (Sete Lagoas = 98,3%) de alfabetizados, e 26,5% (Sete Lagoas = 4,4%) com renda > 20 SMs. O estrato B (médio) com 294 setores (Sete Lagoas = 42), 10,50% SM (Sete Lagoas = 3,18%), 99,9% (Sete Lagoas = 97,3%) e 11,1% (Sete Lagoas = 0,9%), respectivamente. O estrato C (baixo), com 1.091 setores (Sete Lagoas = 69), 5,19% SM (Sete Lagoas = 2,23%), 99,4% (Sete Lagoas = 95,7%) e 2,4% (Sete Lagoas = 0,3%). E o estrato D (muito baixo) com 2.262 setores (Sete Lagoas = 126), 1,91% SM (Sete Lagoas = 1,54%), 94,0% (Sete Lagoas = 92,4%) e 0,2% (Sete Lagoas = 0,1%).

Posteriormente, foram definidos conglomerados com um número mínimo de crianças a serem sorteadas, a fim de se atingir o tamanho da amostra determinada. As crianças foram localizadas a partir das coordenadas geográficas dos endereços disponíveis no Sistema de Informações sobre Nascidos Vivos (Sinasc), e, quando necessário, por busca ativa nos conglomerados. Detalhes da amostragem em cada município foram anteriormente descritos em publicações prévias.^3,10^

### Variáveis de interesse 

O esquema básico completo incluiu o conjunto de vacinas a serem aplicadas durante os primeiros 24 meses de vida e que constam no calendário vacinal básico infantil do Ministério da Saúde: BCG, hepatite B, pentavalente, poliomielite inativada (VIP), pneumocócica 10 valente conjugada, rotavírus humano, meningocócica C conjugada, febre amarela. O esquema completo aos 24 meses inclui, além das vacinas do esquema básico, a tríplice viral (previne sarampo, rubéola e parotidite), hepatite A, varicela e vacina oral de poliovírus atenuados (VOP), e o reforço com tríplice bacteriana (previne difteria, tétano e pertússis), meningocócica C e pneumocócica.^3^ A vacina da febre amarela não foi incluída porque sua introdução no esquema básico variou entre os estados.

As variáveis de exposição foram dados sociodemográficos, maternos (reprodutivos), do domicílio, de consumo da família, e de vacinação da criança, além dos motivos de hesitação vacinal, dificuldades encontradas, e percepções dos responsáveis sobre as vacinas.^3^ Como diferentes composições de vacinas são utilizadas para proteger contra as mesmas doenças e como seu uso é diferente nos setores público e privado, nestas situações, as datas de aplicação dessas vacinas foram padronizadas em uma variável relacionada a cada vacina do calendário do PNI, como, por exemplo, vacina pentavalente = pentavalente + hexavalente + acelular. Detalhes do procedimento para cada vacina foram descritos no relatório técnico do inquérito nacional.^10^

### Fonte de dados/mensuração 

A cobertura, pelo método administrativo, que representa a proporção da população-alvo vacinada, foi obtida pela divisão do número de doses aplicadas de uma vacina pela população-alvo, multiplicada por 100.^11^ No inquérito, a cobertura foi calculada com base nas datas de aplicação das vacinas registradas nas cadernetas de vacinação, tendo os esquemas vacinais sido calculados para doses aplicadas (número de doses registradas de cada vacina) e válidas/oportunas (considerando o momento em que foram aplicadas com relação à data de nascimento e intervalos entre doses).^3^ Esses dados foram obtidos a partir das fotografias da caderneta de vacinação, que foram interpretadas e transcritas por profissionais com experiência em salas de vacina. As crianças sem caderneta foram consideradas não vacinadas após busca sem sucesso no registro no SI-PNI. Demais informações foram obtidas através do questionário aplicado ao responsável pela criança.

Detalhes do trabalho de campo, coleta e transcrição dos dados, bem como problemas observados e potenciais vieses, foram apresentados em publicações anteriores.^3,10^

### Definição e categorização das variáveis 

Características sociodemográficas das famílias:

- Nível de consumo das famílias: definido segundo pontos de corte do Critério de Classificação Econômica Brasil de 2019: alto (42 pontos e mais), médio (27 a 41 pontos), baixo (16 a 26 pontos) e muito baixo (< 16 pontos);^12^
- Aglomeração domiciliar (registrada no questionário domiciliar): presença de mais de três moradores por cômodo usado como dormitório;
- Proporção de famílias contempladas pelo programa Bolsa Família;
- Renda familiar mensal categorizada em faixas: nenhuma renda ou até R$ 300,00, de R$ 301,00 a R$ 1.000,00, de R$ 1.001,00 a R$ 3.000,00, de R$ 3.001,00 a R$ 5.000,00, de R$ 5.001,00 a R$ 8.000,00, mais de R$ 8.000,00.

Características maternas:

- Escolaridade (fundamental incompleto, fundamental completo ou ensino médio incompleto, ensino médio completo ou superior incompleto, ensino superior completo ou mais);
- Faixa etária (< 20 anos, 20 - 34 anos, ≥ 35 anos), raça/cor da pele (branca, parda, preta, amarela, indígena), trabalho remunerado (sim/não), residir com companheiro (sim/não), número de filhos vivos.

Características da criança:

- Sexo (masculino/feminino);
- Ordem do nascimento (primeiro, segundo, terceiro, quarto ou mais);
- Raça/cor da pele (branca, parda, preta, amarela, indígena);
- Frequentar creche (sim, não).

A percepção dos pais em relação às afirmações sobre as vacinas foi avaliada por meio de escala Likert, cuja pontuação das respostas variou de 1 (discorda totalmente) a 5 (concorda totalmente). A pontuação obtida foi posteriormente reagrupada nas seguintes categorias: discordo total ou parcialmente, sou indiferente, concordo total ou parcialmente.^10^ Quanto à hesitação vacinal dos responsáveis pela criança, foram consideradas as concordâncias ou discordâncias em relação às seguintes afirmações: vacinas não são importantes; não confia nas vacinas fornecidas pelo governo; não acredita que as vacinas sejam importantes para a saúde das crianças; vacinas não são importantes para as crianças do bairro; não há necessidade de vacinas para doenças que não existem mais; vacinas causam reações graves.

### Métodos estatísticos 

A análise descritiva das coberturas foi feita separadamente para Belo Horizonte e Sete Lagoas, por meio do cálculo da medidas estatísticas sumárias (médias e proporções) e pela construção de gráficos das estimativas pontuais e intervalares das prevalências de coberturas e demais características da população de estudo, considerando-se os planos amostrais complexos, ponderações das medidas e posterior calibração das amostras populacionais.^3,10^ A interpretação dos resultados foi baseada nas metas de cobertura estabelecidas pelo Ministério da Saúde: 90%, para BCG e rotavírus; 95%, para hepatite B, meningocócica C, pentavalente, pneumocócica, poliomielite, hepatite A, tríplice viral; e de 100%, para a tríplice bacteriana.

As estimativas das características socioeconômicas e demográficas das famílias, mães e crianças do inquérito foram apresentadas apenas para os estratos de renda mais extremos (A e D), devido a similaridades dos níveis de prevalências de cobertura entre os estratos intermediários. As análises foram realizadas no *software* estatístico R versão 4.3.2.^13^

### Considerações éticas

O estudo foi aprovado pelo Comitê de Ética em Pesquisa com Seres Humanos do Instituto de Saúde Coletiva da Universidade Federal da Bahia, sob parecer número 3.366.818, em 04 de junho de 2019, com Certificado de Apresentação de Apreciação Ética (CAAE) 4306919.5.0000.5030; e da Irmandade da Santa Casa de São Paulo, sob parecer número 4.380.019 , em 04 de novembro de 2020, com CAAE 39412020.0.0000.5479. Os entrevistados assinaram o termo de consentimento para a realização da entrevista e a autorização para que a caderneta fosse fotografada.^3^

## RESULTADOS

Em Belo Horizonte, foram estudadas 1.866 crianças (sem perdas), sendo 470 do estrato socioeconômico A, 458 do estrato B, 469 do estrato C e 469 do estrato D. Da amostra total, 99,2% possuíam caderneta de vacinação. Segundo estratos, 99,9%, 98,5%, 99,0% e 99,4% das crianças, respectivamente, dos estratos A, B, C e D, possuíam caderneta. Um percentual de 63,5% das crianças do estrato A e de 9,3% do estrato D foram vacinadas em serviços privados para vacinação. 

Em Sete Lagoas, foram estudadas 451 crianças (sem perdas), das quais 99,9% possuíam caderneta de vacinação, com pequena variação entre os estratos. O percentual de utilização de serviços privados para vacinação foi de 53,7%, no estrato A, e de 19,0%, no estrato D. 

A Tabela 1 apresenta as características socioeconômicas e demográficas das famílias, das mães e crianças do inquérito, segundo os estratos de renda mais extremos (A e D) em Belo Horizonte e Sete Lagoas. Em Belo Horizonte, o percentual de crianças com famílias com consumo alto e médio foi de 70,1%, no estrato A, enquanto o percentual de consumo baixo e muito baixo foi de 87,8%, no estrato D. Em Sete Lagoas, a proporção de famílias com consumo alto e médio no estrato A (53,1%) foi menor do que o observado em Belo Horizonte.

**Tabela 1:** Características sociodemográficas das famílias, mães e crianças incluídas no inquérito segundo os estratos de renda mais extremos, em Belo Horizonte e Sete Lagoas, Inquérito Nacional de Cobertura Vacinal, 2020

Características	Belo Horizonte (%) (n = 1.866)			Sete Lagoas (%) (n = 451)		
	**Estrato A**	**Estrato D**	**Total**	**Estrato A**	**Estrato D**	**Total**
**Características familiares**						
**Nível de consumo familiar**						
Alto	15,3	0,3	3,0	5,2	0,0	1,1
Médio	54,8	12,0	22,1	47,9	9,5	16,5
Baixo	26,0	50,3	45,6	37,3	44,1	44,1
Muito baixo	3,9	37,5	29,3	9,7	46,4	41,4
**Aglomeração intradomiciliar**	0,2	4,6	4,1	1,1	6,8	4,8
**Bolsa família**	6,8	18,2	15,1	7,3	24,9	23,8
**Renda familiar mensal**						
Até R$ 1.000	4,4	30,0	21,7	5,2	25,6	24,9
R$ 1.001 a R$ 3.000	11,0	43,0	37,8	34,1	56,3	46,9
R$ 3.001 a R$ 8.000	21,0	13,5	20,4	22,7	7,7	11,5
Mais de R$ 8.000	32,4	1,6	6,1	26,8	0,0	4,4
Avó morando junto	14,9	32,2	28,1	29,8	23,9	25,4
**Características maternas**						
**Escolaridade**						
Fundamental incompleto	0,7	7,1	6,8	3,9	4,7	4,1
Fundamental comp. /ensino médio incomp.	2,9	14,9	13,0	12,4	12,9	13,2
Ensino médio comp./superior incomp.	19,2	61,4	48,7	21,3	74,1	62,2
Ensino superior completo e mais	76,5	15,0	30,2	62,4	6,1	19,1
**Faixa etária (anos)**						
< 20	0,0	2,2	1,8	0,0	1,1	0,6
20 - 34	28,0	64,7	54,9	34,9	50,2	51,6
≥ 35	72,0	33,1	43,2	65,1	48,6	47,9
**Raça/cor da pele**						
Branca	71,1	52,5	38,8	42,6	9,8	15,6
Parda	20,3	32,4	40,2	5,7	20,1	16,1
Preta	4,8	11,8	14,8	50,5	59,5	60,9
Amarela	2,7	2,0	3,8	1,3	6,3	5,0
Indígena	0,0	0,00	1,2	0,0	4,3	2,4
**Trabalho remunerado**	81,9	44,4	53,5	75,4	37,0	46,9
**Possuir companheiro**	86,7	68,0	70,3	77,1	73,7	68,9
**Número médio de filhos vivos**	1,6	2,0	1,9	1,7	1,9	1,8
**Características da criança**						
**Sexo**						
Masculino	51,1	59,1	56,4	62,7	47,5	53,6
Feminino	48,9	40,9	43,6	37,3	52,5	46,4
**Ordem de nascimento**						
Primeiro	59,6	47,2	51,6	60,3	47,7	50,0
Segundo	33,0	33,0	32,8	25,3	39,5	34,3
Terceiro	6,2	14,9	11,2	13,6	10,7	12,7
Quarto ou mais	1,3	4,9	4,2	0,9	2,2	3,1
**Raça/cor da pele**						
Branca	71,7	40,9	48,9	57,2	33,6	36,5
Parda	22,3	45,4	39,9	1,5	8,6	6,5
Preta	4,4	11,8	9,7	40,9	44,9	50,0
Amarela	1,5	1,0	0,9	0,4	11,1	6,1
Indígena	0,0	0,6	0,3	0,0	1,8	0,9
**Frequenta creche**	62,1	61,6	61,9	42,4	22,7	27,2

Em Belo Horizonte, 53,4% das famílias possuíam renda mensal superior a R$ 3 mil, no estrato A, enquanto em Sete Lagoas, 81,9% do estrato D possuíam renda inferior a esse valor. Em Belo Horizonte e Sete Lagoas, respectivamente, 15,0% e 6,1% das mães do estrato socioeconômico D possuíam ensino superior ou mais, enquanto 76,5% e 62,4% das mães do estrato A possuíam esse nível de escolaridade. Um percentual de 15,1% das famílias, em Belo Horizonte, e 23,8%, em Sete Lagoas, eram beneficiárias do Bolsa Família. 

Mais de 50% das mães tinham entre 20 e 34 anos, moravam com o companheiro (Belo Horizonte: 70,3%; Sete Lagoas: 68,9%) e tinham em média de 1,8 a 1,9 filho, observando-se alta variação na frequência dessas características entre os estratos. Em Belo Horizonte, a maioria das mães se autodeclararam de cor branca (48,9%), enquanto metade das entrevistadas em Sete Lagoas (50,0%) se autodeclaram de cor preta em ambos os estratos. A maioria das mulheres com trabalho remunerado pertencia ao estrato A (Belo Horizonte: 81,9%; Sete Lagoas: 75,4%), de menor vulnerabilidade social. Em relação à ordem de nascimento, cerca da metade das crianças era primogênita (Belo Horizonte: 51,6%; Sete Lagoas: 50,0%). Um percentual de 61,9% e 27,2% das crianças frequentavam creche, em Belo Horizonte e Sete Lagoas, respectivamente.

A Figura 1 mostra as estimativas pontuais das coberturas (%) do esquema completo aos 24 meses de idade, doses aplicadas e válidas, total e segundo características da população, como em ambos os municípios. 

**Figure 1:** Cobertura vacinal (%) do esquema vacinal completo aos 24 meses, segundo características socioeconômicas e demográficas informadas pelo responsável da criança, em Belo Horizonte (n = 1.866) e Sete Lagoas (n = 451), Inquérito Nacional de Cobertura Vacinal, 2020

O município de Sete Lagoas apresentou maior cobertura do esquema completo de doses aplicadas e válidas: 79,1 (IC_95_% 75,0;82,7); 64,9 (IC_95_% 56,9;72,1) do que Belo Horizonte (doses aplicadas: 63,8 (IC_95_% 59,5;67,9); e válidas: 50,2 (IC_95_% 44,1;56,2). Em Sete Lagoas, a cobertura foi maior entre as crianças residentes em domicílios com quatro ou mais moradores, sem diferença entre doses válidas e aplicadas. A cobertura de crianças cujas mães tinham nível de escolaridade fundamental completo foi de cerca de 60% nesse município. 

A Tabela 2 apresenta as coberturas das doses aplicadas e válidas para os imunobiológicos avaliados. Em Belo Horizonte, as coberturas variaram de 75,7%, da dose de reforço da vacina pneumocócica, a 90,3%, para a primeira dose da vacina pentavalente.

**Tabela 2:** Tabela 2 – Cobertura vacinal média estimada e intervalo de confiança de 95% (doses aplicadas e doses válidas) para cada vacina do calendário para os primeiros 24 meses de vida* em Belo Horizonte (n = 1.866) e Sete Lagoas (n = 451), Inquérito Nacional de Cobertura Vacinal, 2020

Vacina	Cobertura (doses aplicadas) % (IC_95_%)		Cobertura (doses válidas) % (IC_95_%)	
	**Belo Horizonte**	**Sete Lagoas**	**Belo Horizonte**	**Sete Lagoas**
BCG	**↓**89,0 (83,3;92,9)	96,3 (88,0;98,9)	**↓**89,0 (83,3;92,9)	96,3 (88,0;98,9)
Hepatite B	**↓**87,9 (82,4;91,9)	96,2 (88,1;98,8)	**↓**87,9 (82,4;91,9)	96,2 (88,1;98,8)
Penta 1^a^ dose	**↓**90,4 (84,6;94,2)	99,2 (98,1;99,7)	**↓**90,3 (84,5;94,1)	99,0 (97,7;99,5)
Penta 2^a^ dose	**↓**89,8 (84,1;93,6)	96,3 (88,1;98,9)	**↓**89,7 (84,1;93,5)	96,3 (88,0;98,9)
Penta 3^a^ dose	**↓**88,6 (83,2;92,4)	**↓**94,5 (87,9;97,6)	**↓**88,1 (82,8;92,0)	**↓**94,2 (87,7;97,4)
Pólio 1^a^ dose	**↓**90,3 (84,6;94,1)	99,2 (98,1;99,7)	**↓**90,2 (84,5;94,0)	98,9 (97,6;99,5)
Pólio 2^a^ dose	**↓**89,7 (84,0;93,5)	96,3 (88,0;98,9)	**↓**89,6 (84,0;93,4)	95,9 (88,2;98,6)
Pólio 3^a^ dose	**↓**88,6 (83,2;92,4)	95,6 (88,3;98,4)	**↓**88,3 (82,9;92,1)	95,3 (88,2;98,2)
Pneumo 1^a^ dose	**↓**90,3 (84,5;94,0)	99,1 (97,9;99,6)	**↓**88,3 (82,4;92,5)	98,1 (96,3;99,0)
Pneumo 2^a^ dose	**↓**89,8 (84,1;93,6)	96,3 (88,1;98,9)	**↓**89,7 (84,1;93,4)	95,9 (88,3;98,6)
Rotavírus 1^a^ dose	**↓**88,8 (83,3;92,7)	94,5 (87,5;97,7)	**↓**88,0 (82,6;91,9)	93,6 (86,9;97,0)
Rotavírus 2^a^ dose	**↓**83,6 (78,4;87,7)	92,3 (86,2;95,8)	**↓**83,2 (78,1;87,3)	92,2 (86,1;95,7)
Meningo C 1^a^ dose	**↓**89,9 (84,3;93,7)	99,3 (98,3;99,7)	**↓**89,2 (83,3;93,2)	99,3 (98,3;99,7)
Meningo C 2^a^ dose	**↓**89,4 (83,8;93,2)	96,2 (88,2;98,8)	**↓**88,8 (83,2;92,6)	**↓**89,4 (84,3;92,9)
Febre amarela	**↓**90,0 (84,3;93,8)	**↓**96,5 (87,9;99,1)	**↓**88,2 (82,5;92,2)	**↓**94,8 (88,0;97,8)
Trip V 1^a^ dose	**↓**89,7(84,0;93,5)	96,7 (87,7;99,2)	**↓**88,9 (83,4;92,7)	96,4 (88,0;99,0)
Trip V 2^a^ dose	**↓**84,9 (79,7;88,9)	**↓**89,4 (83,2;93,5)	**↓**83,7 (78,7;87,7)	**↓**89,1 (83,0;93,2)
Hepatite A	**↓**88,4 (83,0;92,3)	96,1 (88,2;98,8)	**↓**87,7 (82,4;91,6)	**↓**94,5 (88,1;97,5)
Varicela	**↓**88,0 (82,5;91,9)	**↓**92,1 (85,0;95,9)	**↓**84,8 (74,0;91,6)	**↓**91,1 (84,5;95,1)
Pneumo (ref.)	**↓**86,4 (81,3;90,3)	95,0 (88,1;98,0)	**↓**75,7 (71,1;79,8)	**↓**88,2 (82,7;92,1)
Meningo C (ref.)	**↓**83,2 (78,2;87,3)	**↓**93,7 (87,6;96,9)	**↓**78,1 (73,0;82,4)	**↓**87,9 (82,6;91,8)
Pólio (ref.)	**↓**84,2 (78,7;88,5)	95,7 (88,4;98,5)	**↓**81,3 (76,1;85,6)	**↓**91,3 (84,7;95,2)
DTP (ref.)	**↓**82,7 (77,8;86,8)	**↓**93,8 (87,9;96,2)	**↓**82,3 (77,4;86,3)	**↓**83,2 (78,7;87,0)
Esquema completo*	63,8 (59,5;67,9)	79,1 (75,0;82,7)	50,2 (44,1;56,2)	64,9 (56,9;72,1)

BCG: Bacilo de Calmette-Guérin; DTP: Difteria, Tétano e Coqueluche; Meningo C: Meningocócica C; Penta: Pentavalente; Pólio: Poliomielite; Pneumo: Pneumocócica; Trip V: Tríplice viral; Ref.: Dose de reforço; IC_95_%: Intervalo de confiança de 95%; **↓** Coberturas vacinais abaixo do preconizado pelo Ministério da Saúde: acima de 90% (BCG, rotavírus), acima de 95% (hepatite B, pólio, penta, meningo C, hepatite A, tríplice viral, varicela, reforço pólio), acima de 100% (febre amarela, reforço DTP).

A Figura 2 mostra a cobertura das vacinas (doses aplicadas e válidas) do calendário vacinal, preconizadas pelo Ministério da Saúde para crianças com idade até 24 meses, em Belo Horizonte e Sete Lagoas. As cobertura foram menores no estrato A, embora a variabilidade das coberturas tenha sido maior no estrato D nos dois municípios. Em Belo Horizonte, a cobertura de doses válidas foi menor que a de doses aplicadas para o reforço da vacina pneumocócica, sendo maior no estrato A. Em Sete Lagoas, houve maior diferença entre doses aplicadas e doses válidas para o reforço da DTP na população geral, e para o reforço da pneumocócica no estrato A. 

**Figura 2:** Cobertura (doses aplicadas e doses válidas) das vacinas do calendário de crianças com até 24 meses de vida, para o total da amostra e nos estratos socioeconômicos A e D, em Belo Horizonte (n = 1.866) e Sete Lagoas (n = 451), Inquérito Nacional de Cobertura Vacinal, 2020

Quanto à hesitação vacinal, entre as crianças não vacinadas, mesmo na ausência de contraindicações, o percentual de famílias que decidiram pela não vacinação de uma ou mais vacinas foi de 1,6% (IC_95_% 0,9;2,7), em Belo Horizonte, e de 1,0% (IC_95_% 0,3;2,8), em Sete Lagoas. Os maiores percentuais de hesitação, de 1,6% (IC_95_% 0,7;3,6), em Belo Horizonte, e de 1,2% (IC_95_% 0,2;6,1), em Sete Lagoas, ocorreram no estrato D. A dificuldade de acesso ao posto de saúde foi relatada por 7,0% (IC_95_% 4,6;10,5), em Belo Horizonte, e por 6,1% (IC_95_% 3,8;9,5), em Sete Lagoas. Os percentuais de hesitação vacinal nos estratos A e D, respectivamente, foram de 3,5% (IC_95_% 1,1;11,0) e de 8,7% (IC_95_% 5,1;14,5), em Belo Horizonte, e de 5,1% (IC_95_% 2,2;11,0) e 2,6% (IC_95_% 0,9;7,2), em Sete Lagoas. 

Entre os motivos de hesitação vacinal, a não vacinação da criança, mesmo ao levá-la para vacinação no posto de saúde, foi relatada por 18,2% (IC_95_% 14,3;22,8) dos responsáveis em Belo Horizonte. Segundo estratos, a hesitação por esse motivo foi de 12,8% (IC_95_% 6,3;24,2) no estrato A e de 18,9% (IC_95_% 13,3;26,2) no estrato D. Em Sete Lagoas, esse motivo de hesitação foi relatado por 33,1% (IC_95_% 28,2;38,3) dos responsáveis, 34,5% (IC_95_% 22,6;48,7) do estrato A e 30,2% (IC_95_% 25,1;35,8) do estrato D. 

A Figura 3 mostra a frequência dos principais motivos de hesitação vacinal, com destaque para o relato de não vacinação por receio de reações graves, que foi reportado por 18,0% (IC_95_% 14,2;22,4) dos responsáveis pelas crianças, em Belo Horizonte, e por 23,1% (IC_95_% 17,1;30,3), em Sete Lagoas. A hesitação vacinal devido à concepção de que é desnecessário vacinar contra doenças que não mais existiam foi relatada por 16,5% (IC_95_% 13,0;20,6) dos responsáveis entrevistados em Belo Horizonte, e por 10,0% (IC_95_% 5,8;16,7) em Sete Lagoas. A distribuição de frequência dos motivos de hesitação variou entre os estratos econômicos, sendo os motivos de hesitação mencionados acima os mais frequentemente reportados pelos responsáveis de crianças do estrato A, em Belo Horizonte, enquanto a hesitação mais frequentemente reportada no estrato D, em Sete Lagoas, foi o medo de reações adversas.

**Figura 3:** Motivos de hesitação vacinal relatadas pelo responsável de crianças com idade até 24 meses em Belo Horizonte (n = 1.866) e Sete Lagoas (n = 451), total e segundo estratos socioeconômicos A e D, Inquérito Nacional de Cobertura Vacinal, 2020

## DISCUSSÃO

Em geral, as coberturas estiveram abaixo do preconizado, com diferenças significativas entre os estratos socioeconômicos dos municípios. Em Belo Horizonte, todas as vacinas estiveram abaixo das metas, sendo que rotavírus segunda dose e os reforços de DTP e meningocócica C tiveram as menores coberturas com doses aplicadas, e rotavírus segunda dose e os reforços de poliomielite e DTP as menores com doses válidas. Em Sete Lagoas, a tríplice viral segunda dose e os reforços de pneumocócica, meningocócica C e DTP tiveram as menores coberturas. Pentavalente terceira dose, febre amarela, tríplice viral segunda dose, varicela e reforços da meningocócica C e DTP ficaram abaixo das metas. 

Houve maior relato por responsáveis sobre dificuldades de acesso à vacinação em momento oportuno no posto de saúde. Outros motivos da hesitação vacinal citados foram a não vacinação por medo de eventos adversos ou pela crença de que seria desnecessário vacinar a criança contra doenças que não existem mais.

Os resultados sugerem uma boa integração entre os serviços de saúde de imunização em relação ao controle vacinal de crianças, representado pela baixa proporção de crianças sem caderneta de vacinação. Isso pode estar ligado ao fortalecimento do vínculo e a confiança nos serviços de saúde do SUS, bem como por programas sociais, como o Bolsa Família, exigirem que crianças estejam vacinadas, fato que gera maior conscientização sobre a importância da vacinação em comunidades de baixa renda e, consequentemente, maiores coberturas vacinais.^14,15^

As desigualdades socioeconômicas e demográficas na população-alvo dos municípios podem ter impacto na saúde das crianças, afetando as coberturas. Crianças do estrato D têm maior probabilidade de pertencerem a famílias com baixa renda e baixa escolaridade materna, e maior número de filhos, dificultando o acesso a serviços de saúde e comprometendo o seguimento do calendário vacinal. As menores coberturas ocorreram no estrato menos vulnerável (estrato A), apesar de ter havido mudanças nos programas sociais no período, e de os estratos socioeconômicos terem sido definidos com base no Censo de 2010. Além disso, estudos têm mostrado que a conscientização sobre a importância da vacinação é maior em comunidades de baixa renda.^14,15^ Cabe, assim, maior atenção ao estrato A, mesmo se garantindo acesso equitativo às vacinas nos demais estratos, a fim de proteger a saúde e o bem-estar das crianças.^16^

A cobertura foi maior em domicílios com quatro ou mais moradores, o que pode ser explicado por um maior vínculo com serviços básicos de saúde ou de cobertura pela Estratégia Saúde da Família. Em Sete Lagoas, a cobertura foi menor entre crianças de mães com até o ensino fundamental completo, possivelmente pelo menor acesso à informação e educação sobre a importância da imunização. 

O acesso pode ser potencializado com cuidados na atenção primária e a proximidade das famílias com os serviços de saúde, como demonstra a revisão sistemática em países europeus e na Austrália. Em estudo de 2019, mostra-se que aspectos estruturais e organizacionais dos sistemas de cuidado de saúde para crianças pequenas são importantes para a equidade na aceitação das vacinas.^17^

Houve maior hesitação vacinal no estrato A em Belo Horizonte, que pode ser explicada pelo maior acesso à informação e a desinformação, como notícias falsas e boatos de mídias digitais. A dificuldade de acesso a unidade básica de saúde foi o motivo relatado por famílias mais vulneráveis. O medo de reações graves demanda que se trabalhe a divulgação de informações sobre os riscos reais das vacinas, diminuindo as crenças e promovendo maior adesão.^18^ Nesse contexto, os meios digitais amplificam o discurso antivacina, com objeções relacionadas a eventos adversos e minimização da gravidade das doenças.^19^

A revisão sistemática com estudos sobre barreiras à vacinação infantil identificou 573 descrições, codificadas em seis categorias abrangentes: (1) acesso, (2) barreiras clínicas ou do sistema de saúde, (3) preocupações e crenças, (4) percepções e experiências de saúde, (5) conhecimento e informação e (6) influência social ou familiar. Tais motivos apareceram no INCV 2020, sendo necessária uma reflexão sobre estratégias para mudanças do cenário, como campanhas de conscientização sobre a importância da vacinação, foco nos estratos com menores coberturas, capacitação de profissionais da saúde, melhoria do acesso, com atenção especial às famílias em condições de vulnerabilidade social.^20^

Em outra revisão sistemática, foram avaliadas as percepções dos pais, mostrando que consideravam os esquemas de vacinação obrigatórios uma violação dos seus direitos, e não gostavam dos esquemas que ofereciam incentivos financeiros à vacinação. Alguns pais sentiam que os esquemas que limitam o acesso à escola de crianças não vacinadas lhes davam tranquilidade.^21^

No presente estudo, foram descritas coberturas vacinais e informações de hesitação para além dos registros oficiais, com informações dos serviços público e privado, incluindo crianças não vacinadas. Embora possa ter tido impacto no processo de imunização de crianças, os efeitos da pandemia de covid-19 não foram avaliados.^3,10^ Como limitação deste estudo, cita-se a incapacidade de avaliar diferenças significativas entre os municípios avaliados, uma vez que a amostra não foi desenhada para este fim. Ambos os municípios mostraram baixas coberturas e desigualdades entre os estratos socioeconômicos, apontando a necessidade de recuperar altas coberturas, priorizando vacinas com cobertura abaixo do preconizado em todos os estratos socioeconômicos, considerando-se maior acesso e educação em saúde.
